# Effect of canagliflozin treatment on β-cell responsiveness in insulin-dysregulated horses: a 4-week, multi-arm, double-blind, randomized clinical trial

**DOI:** 10.1093/jvimsj/aalag249

**Published:** 2026-10-07

**Authors:** Elin Svonni, Sanna Lindåse, Siv Hanche-Olsen, Ingunn Risnes Hellings, Constanze Fintl, Katarina Nostell, Johan Bröjer

**Affiliations:** Department of Clinical Sciences, Swedish University of Agricultural Sciences, 756 51 Uppsala, Sweden; Department of Clinical Sciences, Swedish University of Agricultural Sciences, 756 51 Uppsala, Sweden; Department of Companion Animal Clinical Sciences, Norwegian University of Life Sciences, 1430 Ås, Norway; Department of Companion Animal Clinical Sciences, Norwegian University of Life Sciences, 1430 Ås, Norway; Department of Companion Animal Clinical Sciences, Norwegian University of Life Sciences, 1430 Ås, Norway; Department of Clinical Sciences, Swedish University of Agricultural Sciences, 756 51 Uppsala, Sweden; Department of Clinical Sciences, Swedish University of Agricultural Sciences, 756 51 Uppsala, Sweden

**Keywords:** equine, equine metabolic syndrome, insulin dysregulation, hyperinsulinemia, sodium-glucose co-transporter 2 inhibitor, SGLT2 inhibitor

## Abstract

**Background:**

The sodium-glucose co-transporter 2 (SGLT2) inhibitor canagliflozin (CAN) decreases hyperinsulinemia in insulin-dysregulated (ID) horses, but its effects on pancreatic β-cell responsiveness to glucose are unknown.

**Hypothesis/Objectives:**

Compare the effects of 2 CAN dosages (0.6 and 1.2 mg/kg) with placebo (PBO) on β-cell responsiveness to IV glucose in ID horses using a graded glucose infusion (GGI) test.

**Animals:**

Forty-two privately-owned ID horses.

**Methods:**

Randomized, multicenter, double-blind, parallel-group, multi-arm, PBO-controlled study. Horses underwent GGI testing before and after 4 weeks of treatment with PO CAN (0.6 or 1.2 mg/kg) or PBO. Indices of β-cell responsiveness included the slope of insulin vs glucose relationship (ΔI vs ΔG) and the ratio of the incremental areas under the curve for insulin and glucose (iAUC_INS_/iAUC_GLU_).

**Results:**

Least squares (LS) means ± SEM ΔI vs ΔG values were lower with CAN (0.6 and 1.2 mg/kg) than with PBO (*P* ≤ .001; 22.0 ± 3.5 and 21.1 ± 3.2 mIU/mmol vs 38.4 ± 3.3 mIU/mmol) with no difference between CAN dosages (*P* = .96). Geometric LS mean (95% CI) iAUC_INS_/iAUC_GLU_ values were lower with CAN (0.6 and 1.2 mg/kg) compared with PBO (*P* ≤ .001; 19.3 [16.5-22.5] and 16.4 [13.9-19.3] mIU/mmol vs 33.1 [28.4-38.7] mIU/mmol).

**Conclusions and clinical importance:**

Canagliflozin decreased β-cell responsiveness to IV glucose in ID horses, representing a key mechanism underlying the hyperinsulinemia-decreasing effect of SGLT2 treatment in horses.

## Introduction

Laminitis is a major welfare concern in horses,^1^^,^^2^ and is associated with hyperinsulinemia.^3^^,^^4^ Despite normal blood glucose concentrations, horses with insulin dysregulation (ID) often exhibit an exaggerated insulin response, which is hypothesized to result, at least in part, from increased pancreatic β-cell secretion.^5^ Although management to decrease hyperinsulinemia can prevent laminitis in many ID horses, some cases require pharmacological treatment.^6^^,^^7^

Glucose is the principal physiological stimulus for insulin secretion from pancreatic β-cells. The intrinsic capacity of the β-cell to sense and respond to changes in blood glucose concentration is most accurately characterized by the dynamic relationship between plasma glucose concentrations and insulin.^8^ The graded glucose infusion (GGI) measures insulin over increasing blood glucose concentrations through stepwise increases in IV glucose infusion rates independent of incretin hormones’ effects. This approach provides a comprehensive assessment of β-cell responsiveness across a wide range of blood glucose concentrations.^9^ Compared with oral glucose tests, the GGI allows more direct evaluation of intrinsic β-cell responsiveness.^10^^,^^11^

Sodium-glucose co-transporter-2 (SGLT2) inhibitors, including canagliflozin (CAN), velagliflozin, dapagliflozin, and ertugliflozin, decrease insulin concentrations in ID horses.^6^^,^^12–15^ This effect is generally attributed to enhanced urinary glucose excretion, which decreases plasma glucose concentration and consequently decreases insulin secretion.^16^ However, additional mechanisms may contribute to the insulin-lowering effects of SGLT2 inhibition.^12^^,^^14^

In a recent study, CAN treatment decreased β-cell responsiveness in ID horses, thereby ameliorating postprandial insulin responses after oral carbohydrate intake.^12^ These findings indicate that modulation of pancreatic β-cell function may contribute to the therapeutic effects of SGLT2 inhibitors beyond the effect of glucosuria. In contrast, 2 studies evaluating a single dose of CAN in healthy horses reported no change in β-cell responsiveness during a GGI.^17^^,^^18^ These inconsistent findings regarding the effect of SGLT2 inhibitors on β-cell responsiveness remain unexplained. Potential explanations include time-dependent effects of SGLT2 inhibitor treatment that are not evident after a single dose, differential effects in horses with hyperinsulinemia compared with those with normal insulin regulation (NIR), or methodological differences between PO and IV glucose challenges.

Therefore, our first objective was to evaluate the effect of CAN treatment at 2 dosages (0.6 or 1.2 mg/kg) on β-cell responsiveness in ID horses using a GGI. Secondary objectives included assessment of the impact of CAN on fasting concentrations of glucose and insulin, and on glucose and insulin response variables during GGI. We hypothesized that CAN treatment would decrease β-cell responsiveness compared with placebo (PBO).

## Materials and methods

A full description of the materials and methods is provided in the Appendix.

### Study design

We used a randomized, double-blind, multi-center, parallel-group, multi-arm, PBO-controlled clinical trial to evaluate the effect of CAN (0.6 or 1.2 mg/kg; Invokana, 100 or 300 mg tablets; Invokana, Multipharma AB) vs PBO on β-cell responsiveness to IV glucose in privately-owned horses and ponies with ID. Treatment was administered PO once daily (approximately 9 pm) by the owners for 4 weeks. Our study was an extension of a previously reported study.^19^

### Enrollment criteria

The inclusion criteria have been described in detail elsewhere.^19^ Briefly, horses older than 2 years with a diagnosis of ID (and insulin concentrations > 100 uIU/mL after an oral sugar test [OST]) were eligible for enrollment. Exclusion criteria included ongoing treatment with therapeutic agents, clinical evidence of ongoing laminitis, systemic diseases, or pregnancy and lactation.

### Experimental protocol

Horses were examined for 5 days during 2 visits 4 weeks apart, with blinded treatment started after the first visit. At each visit, body weight, clinical status, body condition score (BCS),^20^ and cresty neck score (CNS)^21^ were recorded, and an OST (day 3) and a forage-based feed challenge test (day 4) were performed. Here we report on the study extension days. A 240-minute GGI, previously validated for horses,^17^^,^^18^ was performed starting at 7 am on the fifth day of both visits. Glucose was administered IV at stepwise increasing infusion rates of 0.4, 0.8, 1.2, 1.6, 2.4, and 3.2 mg/kg/min, with each infusion step lasting for 40 min. Blood samples were collected throughout the GGI for determination of glucose and insulin concentrations. Plasma insulin was measured using an ELISA^22^ and plasma glucose using an automated clinical chemistry analyzer.^23^

### Outcomes

The primary endpoint was the difference in β-cell responsiveness to IV glucose between CAN (0.6 and 1.2 mg/kg) and PBO. Beta-cell responsiveness was assessed by the slope of the linear regression line of insulin vs glucose concentrations (ΔI vs ΔG) and by the ratio of the incremental areas under the curve for insulin and glucose (iAUC_INS_/iAUC_GLU_) during the GGI.

### Calculations and statistical methods

Primary and secondary endpoints were analyzed using a linear mixed-effects model, including treatment as a fixed effect, baseline (pre-intervention) results as a continuous covariate, and equine hospital as a random effect. Post-hoc multiple comparisons were conducted using the Tukey–Kramer method. Statistical significance was set at *P* < .05.

## Results

### Horses (participants)

The study included 42 horses representing a range of breeds, including British native pony breeds (*n* = 14), Icelandic horses (*n* = 12), Scandinavian native breeds (*n* = 6), crossbreed ponies (*n* = 4), and warmblood and draft breeds, including crossbreeds (*n* = 6). Horses were randomly allocated to one of 3 treatment groups: CAN 0.6 (*n* = 14), CAN 1.2 (*n* = 14), or PBO (*n* = 14). All horses assigned to the CAN-treated groups completed the study. One horse in the PBO group was withdrawn after an accident during transportation after the initial examination. A flow diagram of enrollment is provided in Figure 1. The demographic, clinical, and laboratory characteristics of the participants are summarized in Table 1.

**Figure 1:**
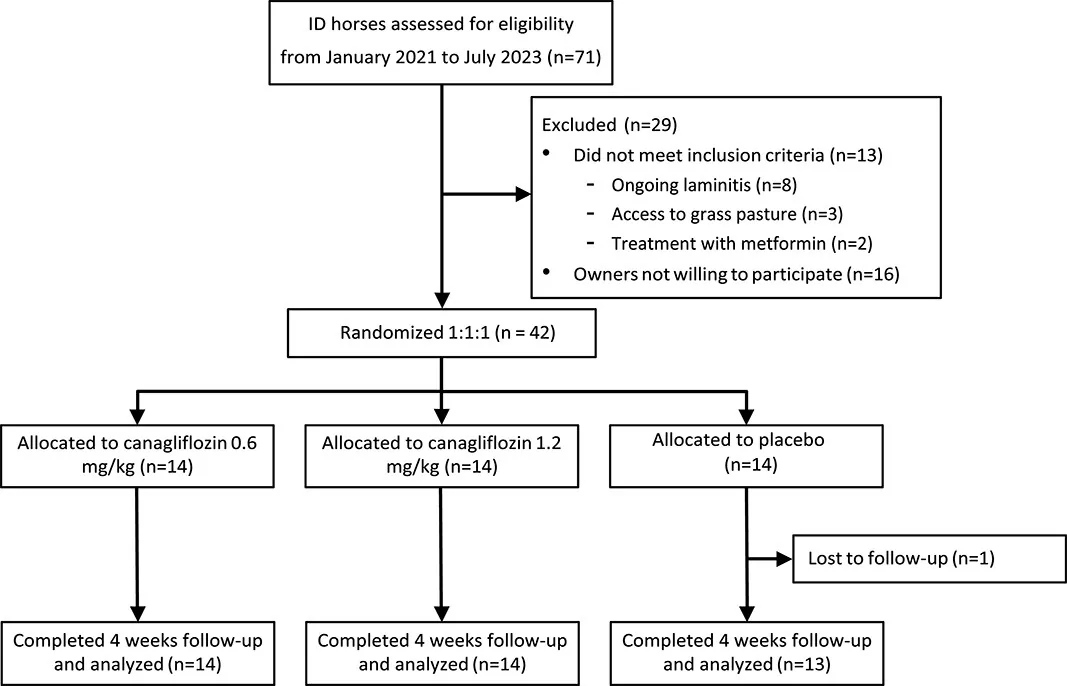
Flow diagram for enrollment, randomization, allocation, follow-up, and data analysis for the 4-week study. Abbreviation: ID, insulin dysregulation.

**Table 1:** Baseline characteristics of participants.

**Variable**	**PBO (*n* = 14)**	**CAN 0.6 (*n* = 14)**	**CAN 1.2 (*n* = 14)**
**Age (years)**	14.9 ± 3.1	12.3 ± 4.5	15.1 ± 3.4
**Sex (mare/gelding)**	11/3	10/4	10/4
**Body weight (kg)**	338 ± 128	381 ± 138	362 ± 93
**BCS**	6.5 (6–8)	7.5 (7-8)	7 (6-8)
**CNS**	3.5 (3–4)	4 (3–4)	4 (3–4)
**Previous laminitis (yes/no)**	13/1	12/2	10/4
**FPG (mmol/L)**	5.1 ± 0.6	5.2 ± 0.4	5.1 ± 0.3
**FPI (μIU/mL)**	35.2 (17.2–63.9)	26.8 (20.7–43.0)	36.7 (28.2–76.3)
**Slope ΔI vs ΔG during GGI (mIU/mmol)**	29.3 (18.7–45.6)	26.6 (14.5–35.5)	39.8 (18.6–62.7)

Values are mean ± SD, median (IQR), n/n (count number), or geometric least squares mean with 95% CI. Abbreviations: BCS, body condition score; CNS, cresty neck score; Slope ΔI vs ΔG, slope of the linear regression line of insulin vs glucose concentrations; CAN, canagliflozin; FPG, fasting plasma glucose; FPI, fasting plasma insulin; GGI, graded glucose infusion; PBO, placebo.

### Responses to GGI

During the GGI tests, plasma glucose concentrations increased progressively with each stepwise increment in glucose infusion rate (Figure 2). The overall glucose response over time, expressed as the area under the curve (AUC_GLU_) and incremental area under the curve (iAUC_GLU_) was significantly lower in horses treated with CAN at both 0.6 mg/kg and 1.2 mg/kg compared with PBO (*P* < .001; Figure 2; Table 2). Specifically, least squares (LS) mean iAUC_GLU_ decreased by 34.2% and 37.4% in the 0.6 mg/kg and 1.2 mg/kg CAN-treated horses, respectively, compared with PBO. Least squares mean differences or LS mean ratios of β-cell responsiveness, response variables, and fasting glucose and insulin concentrations during GGI are reported in Table 3 and pre- and post-intervention values in Supplementary Table S1.

**Figure 2:**
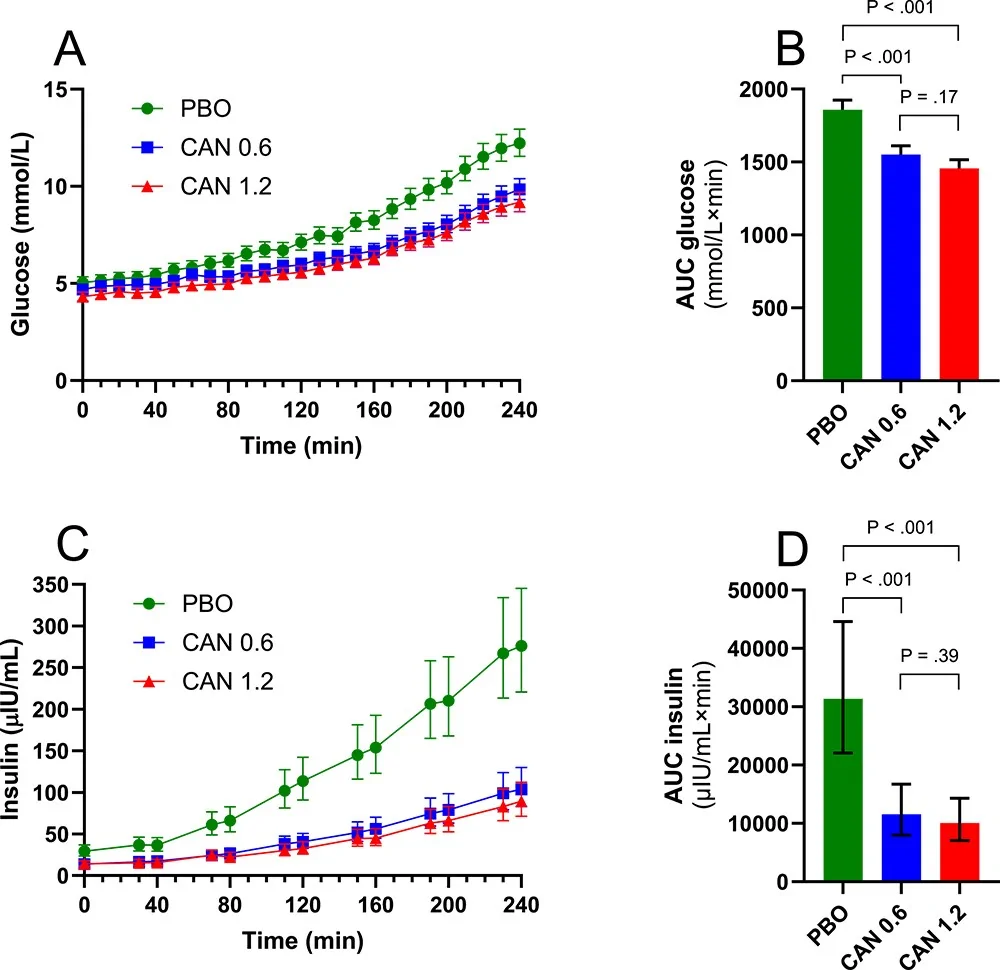
Model-adjusted LS means ± SEM (A and B) or geometric least squares means and 95% confidence intervals (C and D) for concentration-time profiles and AUC of plasma glucose (A and B) and plasma insulin (C and D) in insulin-dysregulated horses treated with CAN (0.6 or 1.2 mg/kg; CAN 0.6 or CAN 1.2) or PBO during a 240-minute GGI. Abbreviations: LS, least squares; AUC, area under the curve; GGI, graded glucose infusion.

**Table 2:** Comparison of primary and secondary endpoints between insulin dysregulated horses treated with either CAN (0.6 or 1.2 mg/kg) or PBO during GGI.

				**Pairwise comparisons (Tukey)**		
**Variable**	**PBO (*n* = 13)**	**CAN 0.6 (*n* = 14)**	**CAN 1.2 (*n* = 14)**	**PBO vs CAN 0.6**	**PBO vs CAN 1.2**	**CAN 0.6 vs CAN 1.2**
**β-cell responsiveness to IV glucose**						
**Slope ΔI vs ΔG (mIU/mmol)**	38.4 ± 3.3	22.0 ± 3.5	21.1 ± 3.2	<.001	<.001	.96
**iAUC**_**INS**_**/iAUC**_**GLU**_ **(mIU/mmol)**	33.1 (28.4–38.7)	19.3 (16.5–22.5)	16.4 (13.9–19.3)	<.001	<.001	.49
**Response variables**						
**iAUC**_**INS**_ **(min***×***mU/L)**	21 567 (18 490–25 156)	7777 (6672–9063)	6408 (5389–7619)	<.001	<.001	.38
**Max**_**INS**_ **(μIU/mL)**	289.4 (241.0–347.6)	103.2 (86.2–123.5)	95.1 (79.2–114.3)	<.001	<.001	.81
**iAUC**_**GLU**_ **(min***×***mol/L)**	644 ± 31	424 ± 32	403 ± 32	<.001	<.001	.82
**Max**_**GLU**_ **(mmol/L)**	12.4 ± 0.3	10.0 ± 0.2	9.0 ± 0.3	<.001	<.001	.06
**Fasting concentrations**						
**FPI (μIU/mL)**	32.0 (10.4–98.1)	14.8 (4.6–47.5)	14.7 (4.7–46.3)	<.001	<.001	1.00
**FPG (mmol/L)**	5.0 ± 0.2	4.7 ± 0.2	4.4 ± 0.2	.19	.01	.20

Means adjusted for baseline (pre-intervention) values are presented as least squares mean ± SEM or geometric least squares means with 95% CI. Abbreviations: Slope ΔI vs ΔG, slope of the linear regression line of insulin vs glucose concentrations; iAUC_INS_, incremental area under the curve for insulin; iAUC_GLU_, incremental area under the curve for glucose; iAUC_INS_/iAUC_GLU_, ratio of incremental area under the curve for insulin and glucose; Max_INS_, maximal insulin concentration; Max_GLU_, maximal glucose concentration; FPI, fasting plasma insulin; FPG, fasting plasma glucose; GGI, graded glucose infusion; CAN, canagliflozin; PBO, placebo.

**Table 3:** Least squares mean differences (D) or least squares mean ratios (R) of β-cell responsiveness, response parameters, and fasting glucose and insulin concentrations during GGI in insulin dysregulated horses treated with PBO, Canagliflozin 0.6 mg/kg (CAN 0.6), or canagliflozin 1.2 mg/kg (CAN 1.2).

**Variable**	**CAN 0.6 vs PBO**		**CAN 1.2 vs PBO**		**CAN 0.6 vs CAN 1.2**	
	**Difference (D) or ratio (R)**	***P*-value**	**Difference (D) or ratio (R)**	***P*-value**	**Difference (D) or ratio (R)**	***P*-value**
**β-cell responsiveness to IV glucose**						
**Slope ΔI vs ΔG (mIU/mmol)**	(D) −16.39 (−25.36 to −7.42)	<.001	(D) −17.34 (−26.19 to −8.49)	<.001	(D) 0.96 (−8.03 to 9.94)	.96
**iAUC**_**INS**_**/iAUC**_**GLU**_ **(mIU/mmol)**	(R) −0.54 (−0.86 to −0.22)	<.001	(R) −0.70 (−1.02 to −0.38)	<.001	(R) 0.16 (−0.18 to 0.50)	.49
**Response parameters**						
**iAUC**_**INS**_ **(min × mU/L)**	(R) −1.02 (−1.33 to −0.71)	<.001	(R) −1.21 (−1.55 to −0.87)	<.001	(R) 0.19 (−0.16 to 0.55)	.38
**Max**_**INS**_ **(μIU/mL)**	(R) −1.03 (−1.34 to −0.72)	<.001	(R) −1.11 (−1.43 to −0.80)	<.001	(R) 0.08 (−0.24 to 0.40)	.81
**iAUC**_**GLU**_ **(min × mol/L)**	(D) −220 (−307 to −134)	<.001	(D) −241 (−329 to 154)	<.001	(D) 21 (−63 to 105)	.82
**Max**_**GLU**_ **(mmol/L)**	(D) −2.43 (−3.47 to −1.39)	<.001	(D) −3.46 (−4.62 to −2.30)	<.001	(D) 1.03 (−0.04 to 2.10)	.06
**Fasting concentrations**						
**FPI (μIU/mL)**	(R) −0.77 (−1.19 to −0.35)	<.001	(R) −0.78 (−1.20 to −0.35)	<.001	(R) 0.01 (−0.42 to 0.43)	1.00
**FPG (mmol/L)**	(D) −0.33 (−0.79 to 0.13)	.19	(D) −0.66 (−1.11 to −0.20)	0.004	(D) 0.32 (−0.13 to 0.77)	.20

Abbreviations: Slope ΔI vs ΔG, slope of the linear regression line of insulin vs glucose concentrations; iAUC_INS,_ incremental area under the curve for insulin; iAUC_GLU_, incremental area under the curve for glucose; iAUC_INS_/iAUC_GLU_, ratio of incremental area under the curve for insulin and glucose; Max_INS_, maximal insulin concentration; Max_GLU_, maximal glucose concentration; FPI, fasting plasma insulin; FPG, fasting plasma glucose; GGI, graded glucose infusion; CAN, canagliflozin; PBO, placebo.

Glucose-stimulated insulin secretion (GSIS) increased progressively across all treatment groups, as reflected by increasing plasma insulin concentrations during the GGI. The overall insulin response over time (AUC_INS_) was lower in both CAN-treated groups compared with PBO (*P* < .001; Figure 2). The incremental insulin response over time (geometric LS mean iAUC_INS_) decreased by 63.9% and 70.3% in CAN-treated horses receiving CAN 0.6 and CAN 1.2, respectively, indicating a substantial suppression of GSIS after CAN treatment.

### Dose–response relationship

The slopes of the linear regression lines of insulin vs glucose concentrations (ΔI vs ΔG) during GGI were computed for all horses, and adjusted means for each treatment group are presented in Table 2. The median R^2^ for the linear regression analyses for all horses was 0.97 (IQR, 0.94-0.98; *P* ≤ .006 before treatment and 0.97, (IQR, 0.95-0.99; *P* ≤ .06) after treatment. The overall dose–response relationships between LS mean plasma glucose and LS geometric mean insulin concentrations at the end of each infusion step during the GGI for horses receiving CAN 0.6 mg/kg, CAN 1.2 mg/kg, and PBO are presented in Figure 3. For each treatment group, the plotted adjusted means approximate a linear relationship. The plotted data for CAN (0.6 and 1.2 mg/kg) treated horses are shifted leftward and demonstrate a narrower range of glucose concentrations compared with PBO-treated horses. These findings correspond to significantly lower fasting plasma glucose concentration (FPG) in horses receiving 1.2 CAN (*P* = .01) as well as decreased Max_GLU_ in CAN-treated (0.6 and 1.2 mg/kg) horses (*P* ≤ .001) compared with PBO (Table 2). Furthermore, the data for CAN-treated horses are shifted downwards and follow less steep slopes than those of the PBO group, indicating a lower dose–response relationship between plasma glucose and plasma insulin concentrations with CAN treatment. The combination of a lower dose–response relationship and narrower range of glucose concentrations with CAN treatment is reflected by significantly lower fasting plasma insulin concentrations (FPI) and Max_INS_ (*P* ≤ .001) in horses treated with CAN (0.6 and 1.2 mg/kg) compared with PBO (Table 2).

**Figure 3:**
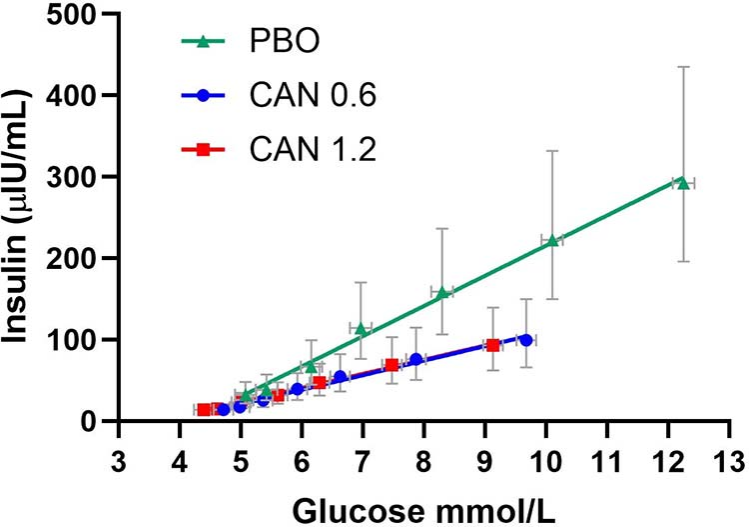
Dose–response relationship between plasma glucose (least squares means ± SEM) and plasma insulin (geometric least squares means with 95% confidence interval) concentrations during the GGI test in horses receiving placebo (PBO, triangle), CAN 0.6 mg/kg (circle), and CAN 1.2 mg/kg (square). Data at 0, 40, 80, 120, 160, 200, and 240 min were derived from a linear mixed-effects model for repeated measures over time for GGI glucose and insulin data, respectively. The adjusted mean pairs (insulin vs glucose) for each group of treated horses follow a straight line.

## Discussion

The effect of SGLT2 inhibitor treatment on β-cell responsiveness (the degree of insulin response to a given plasma glucose concentration) has not been well investigated in horses. Our study showed that CAN significantly decreased β-cell responsiveness to IV glucose in ID horses, with no detectable difference between the 2 dosages evaluated. These findings align with a previous study in ID horses in which CAN decreased indices of β-cell responsiveness during PO glucose administration.^12^ Additionally, several studies in ID horses treated with SGLT2 inhibitors have reported a proportionally larger decrease in insulin than glucose responses during a PO glucose challenge, although indices of β-cell responsiveness were not calculated.^6^^,^^13^^,^^24^ In contrast, in a separate study, horses with NIR receiving a single dose of CAN did not exhibit decreased β-cell responsiveness during a GGI.^17^^,^^18^ Collectively, these results suggest that CAN may affect β-cell responsiveness differently in horses with hyperinsulinemia compared with those with NIR, or that repeated dosing may be required to achieve this effect.

Although β-cell responsiveness is rarely measured directly in studies of horses, it has been hypothesized to be increased in ID horses and to contribute to excessive GSIS.^25^ However, GSIS is influenced by several factors, and there is limited knowledge about the extent to which increased β-cell responsiveness contributes to hyperinsulinemia in ID horses. Most research has focused on the exaggerated insulin responses after PO administration of glucose or carbohydrates, which is thought to involve an increased incretin response in ID horses.^26–28^ In our study, the adjusted mean β-cell responsiveness for the PBO-treated horses, expressed as the slope of ΔI vs ΔG, was 38.4 mIU/mmol. In comparison, the mean β-cell responsiveness reported for healthy PBO-treated Icelandic horses in a separate study, which used a similar GGI protocol and identical analytical methods for insulin and glucose, was 14.3 mIU/mmol.^18^ In another study investigating β-cell responsiveness in healthy Standardbred trotters using a hyperglycemic clamp, the mean of the slope of ΔI vs ΔG was 10.1 mIU/mmol.^29^ This indicates the β-cell responsiveness of horses with ID in our current study to be 2.6 to 3.7 times higher than horses with NIR reported in a separate study. These findings support the hypothesis that increased β-cell responsiveness is an important contributor to hyperinsulinemia in horses, occurring independently of the intestinal incretin effects. Taken together, these results suggest that blood glucose is a primary driver of insulin secretion in horses with ID, whereas incretin-mediated mechanisms appear to make a comparatively smaller contribution.

The marked decrease in insulin concentrations observed in CAN-treated horses may partly reflect a direct response to lower plasma glucose concentrations secondary to increased urinary glucose excretion, the primary mechanism of action of SGLT2 inhibitors. Nonetheless, insulin concentrations decreased proportionally more than plasma glucose concentrations, which are primarily decreased by increased urinary glucose excretion during the GGI (Figure 3), suggesting that factors other than glucose lowering may contribute to the observed suppression of insulin secretion. The horses in our current study received an IV glucose load. Therefore, any effects of CAN treatment on the entero-insular axis and intestinal incretin hormone secretion can be excluded as contributing factors to the observed decrease in β-cell responsiveness. Additionally, horses were off feed for 8 hours before the start of the GGI, making any possible residual incretin effects on insulin secretion unlikely.

A larger decrease in insulin concentrations compared with glucose concentrations may result from increased insulin sensitivity with a secondary decrease in β-cell responsiveness, or from a primary decrease in β-cell responsiveness with or without altered insulin sensitivity. It is possible that CAN treatment increased insulin sensitivity in our current study, resulting in enhanced insulin-mediated glucose disposal in peripheral tissues and a compensatory decrease in β-cell responsiveness. Despite some conflicting results, SGLT2 inhibitors have been reported to improve insulin sensitivity in animal models and in humans with type 2 diabetes (T2D). Proposed mechanisms include decreased glucotoxicity in skeletal muscle cells and weight loss.^30–34^ Decreased glucotoxicity is a less likely explanation in ID horses with nearly normal plasma glucose concentrations. Nevertheless, short-term SGLT2 inhibitor treatment has been shown to induce weight loss in horses.^7^^,^^12^ In the original study, of which our present study is an extension, body weight decreased by 2.9% (CAN 0.6) and 3.9% (CAN 1.2) compared with PBO during the 4-week treatment period (results presented elsewhere).^19^ Studies evaluating the effect of weight loss on insulin sensitivity in horses are limited and have yielded inconclusive results.^35–37^ Substantial weight loss, addition of an exercise program, or both are likely necessary to achieve clinically meaningful improvements in insulin sensitivity in horses.^36^^,^^38^^,^^39^ The modest weight loss observed in the CAN-treated horses in our current study may have contributed to some improvement in insulin sensitivity. However, a pronounced effect attributable to weight reduction seems unlikely, although increased insulin sensitivity mediated by other mechanisms cannot be excluded.

Another potential mechanism underlying the decrease in β-cell responsiveness, occurring over time rather than after a single dose of CAN, is adaptation to sustained decreases in plasma glucose concentration secondary to urinary glucose excretion. The amount of urinary glucose excretion was not measured in our current study because of practical constraints, and thus the amount of glucose diverted from stimulating β-cell insulin secretion is unknown. Given that CAN is among the less selective SGLT2 inhibitors,^40^ additional glucose-lowering effects mediated through SGLT1 inhibition in the renal and gastrointestinal tracts cannot be excluded in clinical cases. However, in our current study, in which responses to an IV challenge were assessed, effects mediated via SGLT1 in the gastrointestinal tract are unlikely to have contributed to the glucose-lowering effects.

Chronic decreases in plasma glucose concentration may induce a decrease in β-cell responsiveness and decreased GSIS. However, as mentioned earlier, horses with ID generally have normal to only mildly increased plasma glucose concentrations. It therefore remains uncertain whether a modest decrease in glucose concentrations would be sufficient to explain the pronounced decrease in β-cell responsiveness observed in the study.

The expression of SGLT2 in β-cells, as well as any direct pancreatic effects of SGLT2 inhibitors, has not been demonstrated in other species,^41^^,^^42^ and it remains uncertain whether a direct pancreatic effect influencing β-cell responsiveness exists in horses. A direct pharmacologic effect on β-cells would be expected to occur after a single dose of SGLT2 inhibitors. In contrast, studies in healthy horses receiving a single dose of CAN found no change in β-cell responsiveness,^17^^,^^18^ arguing against an immediate direct effect. Nevertheless, although less likely, it is possible that such an effect occurs selectively in horses with hyperinsulinemia, but not in those with NIR.

In contrast to the effect observed in the horses in our current study, treatment with SGLT2 inhibitors has been shown to enhance β-cell responsiveness to IV glucose in humans with T2D.^43^^,^^44^ However, these populations differ substantially, and horses with ID may more closely resemble humans with metabolic syndrome or prediabetes, conditions characterized by mild hyperglycemia and often, although seldom measured, hyperinsulinemia.^45^^,^^46^ Individuals with metabolic syndrome or prediabetes are rarely treated with SGLT2 inhibitors, and data on the effects of such treatment in these populations remain limited. However, studies of humans with obesity and insulin resistance but without diagnosed T2D have demonstrated that treatment with SGLT2 inhibitors substantially decreased the insulinogenic index (ΔAUC_INS_/ΔAUC_GLU_). ^47^^,^^48^ The insulinogenic index is a measure of the early insulin response to glucose during a PO glucose tolerance test and is commonly used to evaluate β-cell responsiveness.^11^ Although these effects were observed during PO challenges, they parallel the decreased β-cell responsiveness observed in our current study and suggest cross-species similarities. Furthermore, several studies in animal models and in humans with hyperinsulinemia have reported a decreased insulin response or decreased FPI concentration after SGLT2 inhibitor treatment, although β-cell responsiveness was not directly measured.^32^^,^^34^^,^^49^ Similar responses in insulin secretion observed in humans with metabolic syndrome characterized by insulin resistance and in horses with ID support the hypothesis that insulin resistance may be an important component of ID in horses.

No significant differences were observed in response variables or fasting concentrations between the 2 CAN doses (0.6 and 1.2 mg/kg) evaluated in our study. One potential explanation is that the lower dose was sufficient to achieve a near-maximal inhibitory effect on renal SGLT2 activity. Alternatively, the absence of significant differences between the 2 dosing regimens may reflect limited statistical power because of the small sample size of horses. The PBO-treated horses demonstrated an increase in indices of β-cell responsiveness over the 4-week treatment period (Supplementary Table S1). This finding most likely reflects biological and methodological variability associated with repeated metabolic testing rather than a true treatment-independent change in β-cell function. For example, differences in stress levels or sympathetic activation between examinations may have influenced insulin secretion during the second GGI, but these factors were not evaluated in our current study. Regardless of the underlying cause, the inclusion of a PBO-treated control group strengthened the internal validity of the trial by allowing treatment-related effects to be distinguished from temporal variation and other nonspecific influences.

Our study had some limitations. The sample size was limited, and enrolled horses represented multiple breeds, which may have increased variability within the treatment groups. The treatment period was short, and it cannot be excluded that the effect on β-cell responsiveness may differ with longer treatment duration. To differentiate between decreased insulin secretion and increased insulin clearance as potential explanations for the decreased ΔI vs ΔG slope observed, measurement of C-peptide concentrations would have been informative. Insulin and C-peptide are secreted from pancreatic β-cells in a 1:1 ratio, but a substantial portion of insulin undergoes first-pass hepatic metabolism before reaching the systemic circulation, whereas C-peptide bypasses hepatic clearance.^50^^,^^51^ Therefore, measurement of C-peptide concentrations provides a more accurate estimate of β-cell responsiveness than plasma insulin concentrations and allows indirect assessment of insulin clearance. The effects of SGLT2 inhibitors on insulin clearance have not been investigated in horses. Evidence from other species is inconsistent, with reports of increased or unchanged clearance after SGLT2 inhibitor administration.^49^^,^^52^ At the time our study was conducted, no validated assay for equine C-peptide was available. Consequently, it cannot be excluded that CAN treatment caused increased insulin clearance, thereby contributing to the decreased insulin concentrations observed, thus potentially leading to an overestimation of the treatment effect on β-cell responsiveness. Nevertheless, estimates of β-cell responsiveness using the slope of insulin vs glucose concentrations correlate well with gold standard measures based on insulin secretion rates derived from C-peptide deconvolution.^53^

In conclusion, CAN treatment significantly decreased pancreatic β-cell responsiveness to IV glucose in ID horses, with no detectable difference between the 2 administered doses. The attenuated insulin response observed during IV glucose infusion in the GGI protocol appears to result from both decreased glycemic stimulation and a direct decrease in β-cell responsiveness. The precise mechanism underlying the decrease in β-cell responsiveness after CAN administration remains to be elucidated.

## Supplementary Material

Supplementary_material_aalag249

## Off-label antimicrobial declaration

Authors declare no off-label use of antimicrobials.

## Institutional animal care and use committee or other approval declaration

The study protocol was approved by the Ethics Committees in Sweden (Dnr: 5.8.18-09082-2020) and Norway (FOTS ID 123285).

## Appendix

### Materials and methods

#### Study design

The study was a randomized, double-blind, multi-center, parallel-group, multi-arm, PBO-controlled clinical trial in which privately-owned horses and ponies (hereafter collectively referred to as horses) diagnosed with ID were randomly assigned to receive 1 of 2 different doses of CAN (Invokana, 100 or 300 mg tablets; Invokana, Multipharma AB) or PBO, administered PO once daily for 4 weeks (28 days).

Briefly, the included horses underwent two 5-day examination periods at the Equine Hospitals at the Swedish University of Agricultural Sciences and at the Norwegian University of Life Sciences separated by a 24-day double-blind treatment period conducted in their home environment. Each examination period consisted of 2 initial days of acclimatization, followed by an OST on day 3, a feed challenge test on day 4 and a 240-minute GGI in the morning on day 5. The second 5-day examination period contained double-blind treatment. The horse owners were instructed to maintain the horse’s diet, exercise, and turnout time constant for 2 weeks before and during the study period. All horse owners provided written informed consent.

#### Enrollment criteria

The inclusion and exclusion criteria have been described in detail elsewhere.^19^ A flow chart of enrollment is provided in Figure 1. Briefly, privately-owned horses older than 2 years previously diagnosed with ID (insulin concentration exceeding 100 μIU/mL; Equine Insulin ELISA, Mercodia AB) based on an OST (Dan Sukker Glykossirap, Nordic Sugar A/S; 0.2 mL/kg body weight) were enrolled in the study. Horses were initially assessed by field veterinarians, who also performed the OST before enrollment. Horses treated with any type of therapeutic agents including supplements with potential effects on insulin metabolism or with clinical evidence of an ongoing episode of laminitis, pituitary pars intermedia dysfunction including assessment of adrenocorticotropic hormone concentrations adjusted for season, or other systemic diseases were excluded. Pregnant or lactating mares also were excluded from the study. A lameness examination specifically focusing on clinical evidence of laminitis was performed by referring veterinarians before enrollment and by staff involved in the research project when horses arrived at the equine hospitals for each 5-day examination period.

#### Randomization and blinding

The horses were randomized in a 1:1:1 ratio to 3 treatment groups (once daily PO treatment with CAN 0.6 or 1.2 mg/kg, or PBO). The detailed procedure for the preparation of tablets and blinding of treatment were performed as previously described.^12^ Briefly, because PBO tablets identical to the active drug were not available, lactose powder dispensed in plastic containers identical to those used for the active drug was used. Lactose powder was chosen because it is a compound in the CAN tablets. The primary containers were packed and dispensed in identical secondary boxes for both treatments. Treatment allocation was blinded to investigators, horse owners and staff at the equine hospitals. One personnel member in each country dispensed the drug and PBO, provided medication to the hospital staff, and responded to questions regarding the treatment.

#### Experimental protocol

The experimental protocol is described in detail elsewhere.^19^ Briefly, horses were examined during two 5-day visits at the University Equine Hospitals in Sweden and Norway. Baseline data were obtained at the first visit, and a follow-up examination was performed after 4 weeks of treatment. Horses were housed at their home stables between examination periods and transported to the equine clinic by their owners before each examination period. Double-blind treatment started when the horses had been transported back to their home stables after the first visit (the same day the GGI was conducted) and continued over the follow-up period. Study medication was administered by the owners at the home stables between the examination periods and by equine clinic staff during the examination periods, daily at 9 PM, 1 h before the final feeding of the day. During both visits, horses were housed in individual stalls with daily access to a sand paddock and were fed their own regular diet divided into 4 meals per day. After transport to the equine clinic, horses were allowed to acclimatize to the environment for 2 days before the first examination. On the second day of the visits, an IV catheter for blood sampling was inserted in one of the jugular veins under topical local anesthesia. Body weight was measured; a clinical examination was performed and BCS^20^ and CNS^21^ were obtained. In the main study, an OST (glucose syrup, 0.5 mL/kg body weight) was performed on day 3. A feed challenge test using each horse’s regular forage (0.4 kg dry matter/kg body weight) was performed on day 4 (data presented elsewhere^19^). On day 4, after completion of the feed challenge test, an additional IV catheter was inserted in the contralateral jugular vein. Serum samples for biochemistry analysis including triglycerides and liver enzyme activities, were obtained in the morning before the feed challenge test, both before and after the intervention. The results are reported elsewhere.^19^ During the study, owners were asked to record the horses’ general attitude, appetite and urine volume on a weekly basis, as well as report any adverse effects to the investigators. These results are reported elsewhere.^19^

For the extended study presented here (day 5), a 240-min GGI tailored for use in horses^18^ was started at 7 a.m. on the fifth day of both clinical visits. Feed was withheld for 8 h overnight. In the morning, the horse’s weight was measured, and a baseline blood sample was obtained before the start of the infusion. An infusion with glucose (Glucose Fresenius Kabi 200 mg/mL, Fresenius Kabi) was administered through one of the indwelling IV catheters at an initial infusion rate of 0.4 mg/kg/min. The glucose infusion rate was increased every 40 min to 0.8, 1.2, 1.6, 2.4 and 3.2 mg/kg/min. After 240 min, the infusion was stopped, the IV catheters were removed, and the horses were fed.

Blood samples for glucose analysis were collected every 10 min throughout the infusion period, whereas insulin samples were obtained 10 min apart during the last 20 min of each infusion step. Blood samples were collected from the indwelling IV catheter not used for glucose infusion and blood was transferred to vacutainer tubes containing lithium heparin. The samples were centrifuged within 5 min; plasma was harvested and immediately frozen at −80°C. Plasma insulin concentrations were analyzed in duplicate using an ELISA optimized for horses (Equine Insulin ELISA; Mercodia AB).^22^ Plasma glucose concentrations were analyzed as single samples using an automated clinical chemistry analyzer (YSI 2500 glucose/lactate analyzer; Yellow Springs Instruments).^23^

#### Outcomes

The primary endpoint was the difference in β-cell responsiveness to IV glucose between 2 different dosages of CAN (0.6 and 1.2 mg/kg) and PBO. Beta-cell responsiveness was expressed as the slope of the linear regression line of insulin vs glucose concentrations (ΔI vs ΔG) and as the ratio of the incremental area under the curve for insulin and glucose (iAUC_INS_/iAUC_GLU_), during a GGI. Secondary endpoints included treatment-related differences in fasting and response variables during a GGI and included FPI and FPG concentrations, AUC and iAUC for insulin and glucose, as well as maximum insulin (Max_INS_) and glucose (Max_GLU_) concentrations.

#### Calculations and statistical methods

JMP Pro version 19.0.0 (SAS Institute Inc) was used to perform all statistical analyses. The AUC_GLU_ and AUC_INS_ were calculated from GGI data using the trapezoidal method. Incremental AUC (iAUC_GLU_ and iAUC_INS_) was derived by subtracting the area of fasting concentrations over time (fasting concentration *×* 240 min) from the corresponding total AUC. The ratio of iAUC_INS_/iAUC_GLU_ was calculated as an index for β-cell responsiveness to IV glucose. A second index of β-cell responsiveness to changes in IV glucose (ΔI vs ΔG) was obtained by simple linear regression of insulin vs glucose concentrations measured at fasting and at the end of each glucose infusion step during each GGI. The slope variable (ΔI vs ΔG) of the linear relationship provides an index of the ability of the β-cell to sense changes in prevailing glucose concentrations and then adapt and respond with appropriate changes in insulin secretion (Supplement Figure S1).

Maximum glucose (Max_GLU_) and maximum insulin (Max_INS_) concentrations were obtained from GGI data.

The primary and secondary endpoints were analyzed using a linear mixed-effects model, with treatment as fixed effect, the corresponding baseline (pre-intervention) as continues covariate to adjust for baseline results and equine hospital as a random effect. The initial full model included a treatment-by-baseline interaction term, but the interaction term was never significant, and it therefore was excluded from the final models.

Glucose and insulin responses over time during the GGI after treatment were analyzed using mixed-effects models with treatment and sampling time point as fixed effects and pre-treatment log-transformation AUC_GLU_ or AUC_INS_ as continuous covariates. Equine hospital and horse nested in equine hospital were included as random effects.

Residual vs predicted plots and Q-Q plots were performed to evaluate assumptions of equal variance and normal distribution, respectively. When necessary, log transformation was applied to address violations of assumptions. Adjusted means for normally distributed data are reported as LS means ± SEM and the LS mean differences between treatments are reported with 2-sided 95% confidence intervals. For log-transformed data, results are reported as geometric LS means and 95% CI after back-transformation to the original scale. Least squares mean differences cannot be reported for log-transformed data after back-transformation. Instead, LS mean differences are reported as geometric LS mean ratios (expressed as percentage) with 2-sided 95% CIs after back transformation.^12^

Tukey–Kramer post-hoc test was applied for multiple comparisons between treatments. Values where *P* < .05 were considered significant.

## Abbreviations

AUC_GLU_: area under the curve for glucose
AUC_INS_: area under the curve for insulin
BCS: body condition score
CNS: cresty neck score
CV: coefficient of variation
ΔI vs ΔG: slope of the linear regression line of insulin vs glucose concentrations
FPG: fasting plasma glucose concentration
FPI: fasting plasma insulin concentration
GGI: graded glucose infusion
GSIS: glucose-stimulated insulin secretion
iAUC_GLU_: incremental area under the curve for glucose
iAUC_INS_: incremental area under the curve for insulin
iAUC_INS_/iAUC_GLU_: ratio of incremental area under the curve for insulin and glucose
ID: insulin dysregulation
LS means: least squares means
Max_GLU_: maximal glucose concentration
Max_INS_: maximal insulin concentration
NIR: normal insulin regulation
OST: oral sugar test
PBO: placebo
SEM: standard error of the mean
SGLT2: sodium-glucose co-transporter 2
T2D: type 2 diabetes
