# Supplementary material for: Effect of canagliflozin treatment on β-cell responsiveness in insulin-dysregulated horses: a 4-week, multi-arm, double-blind, randomized clinical trial
Source: J Vet Intern Med. 2026 Oct 7;40(5):aalag249. doi: 10.1093/jvimsj/aalag249 (PMC13644825; doi:10.1093/jvimsj/aalag249)
Supplement: Supplementary file 1 [file supplementary_material_aalag249.zip › Supplementary Table S1 JVIM 2026-09 .pdf]

Supplementary Table S1. Baseline (pre-treatment) and post-treatment values for horses with insulin dysregulation treated with canagliflozin (0.6 or 1.2 mg/kg) or placebo for 4 weeks.

	**Basal values (n=42)**	**Post treatment values**		
**Variable**		**Placebo (n=13)**	**Canagliflozin 0.6 (n=14)**	**Canagliflozin 1.2 (n=14)**
**β-cell responsiveness to IV glucose**				
Slope ΔI vs. ΔG (mIU/mmol)	28.7 (18.5 – 47.4)	41.9 (19.7 – 55.2)	12.7 (10.3 – 20.4)	20.9 (10.6 – 30.8)
$iAUC_{INS}/iAUC_{GLU}$ (mIU/mmol)	30.2 (20.9 – 54.4)	41.5 (20.8 – 49.8)	14.6 (10.4 – 22.6)	17.5 (11.3 – 34.8)
**Response variables**				
$iAUC_{INS}$ (min×mU/L)	20309 (13011 – 29708)	20824 (14094 – 28180)	6055 (4686 – 8940)	8272 (4528 – 13330)
Max_{INS} (µIU/mL)	254.0 (171.4 – 373.6)	290 (196.7 – 360.4)	81.7 (65.5 – 122.6)	139.0 (61.5 – 189.6)
$iAUC_{GLU}$ (min×mol/L)	659 ± 131	624 ± 147	428 ± 89	412 ± 89
Max_{GLU} (mmol/L)	12.3 ± 1.6	12.2 ± 2.2	10.0 ± 0.7	9.3 ± 0.6
**Fasting concentrations**				
FPI (µIU/mL)	31.1 (20.9 – 60.9)	28.3 (12.0 – 72.4)	11.7 (9.6 – 17.5)	16.8 (9.4 – 26.3)
FPG (mmol/L)	5.1 ± 0.5	5.0 ± 0.4	4.7 ± 0.4	4.4 ± 0.7

Values are mean ± standard deviation or median (interquartile range). Abbreviations: Slope ΔI vs. ΔG, slope of the linear regression line of insulin vs. glucose concentrations; $iAUC_{INS}$, incremental area under the curve for insulin; $iAUC_{GLU}$, incremental area under the curve for glucose; $iAUC_{INS}/iAUC_{GLU}$, ratio of incremental area under the curve for insulin and glucose; Max_{INS}, maximal insulin concentration; Max_{GLU}, maximal glucose concentration; FPI, fasting plasma insulin; FPG, fasting plasma glucose.
