# Supplementary material for: Effect of canagliflozin treatment on β-cell responsiveness in insulin-dysregulated horses: a 4-week, multi-arm, double-blind, randomized clinical trial
Source: J Vet Intern Med. 2026 Oct 7;40(5):aalag249. doi: 10.1093/jvimsj/aalag249 (PMC13644825; doi:10.1093/jvimsj/aalag249)
Supplement: Supplementary file 1 [file supplementary_material_aalag249.zip › Supplementary Figure S1 JVIM 2026-06.pdf]

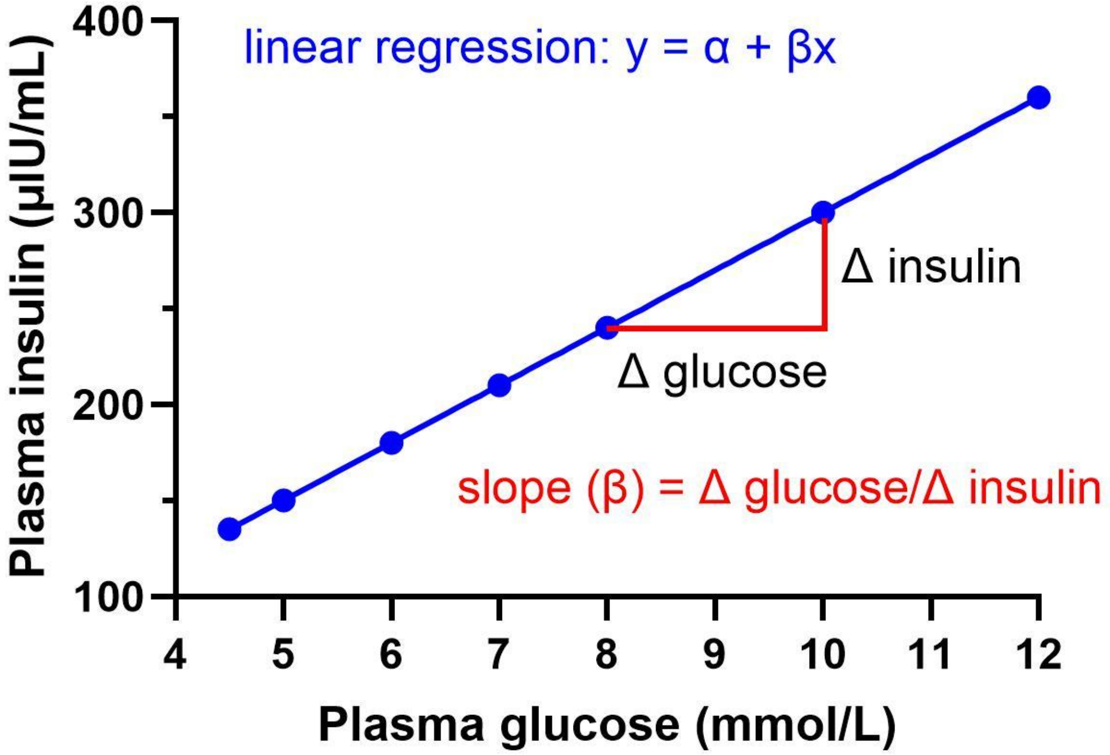

Supplementary Figure S1. The slopes (β) from the simple linear regression of insulin vs. glucose concentrations (Δ glucose/Δ insulin) during graded glucose infusion (GGI) were computed for all horses. The slope parameter of the linear relationship provides an index of the ability of the β-cell to sense changes in prevailing glucose concentrations and then adapt and respond with appropriate changes in insulin secretion.
